# Sub‐Micron Replication of Fused Silica Glass and Amorphous Metals for Tool‐Based Manufacturing

**DOI:** 10.1002/advs.202405320

**Published:** 2024-07-12

**Authors:** Sebastian Kluck, Richard Prediger, Leonhard Hambitzer, Niloofar Nekoonam, Franziska Dreher, Manuel Luitz, Markus Lunzer, Matthias Worgull, Marc Schneider, Bastian E. Rapp, Frederik Kotz‐Helmer

**Affiliations:** ^1^ Laboratory of Process Technology NeptunLab Department of Microsystems Engineering (IMTEK) University of Freiburg 79108 Freiburg im Breisgau Germany; ^2^ UpNano GmbH Modecenterstrasse 22/D36 Vienna 1030 Austria; ^3^ Institute of Microstructure Technology (IMT) Karlsruhe Institute of Technology (KIT) H.‐v.‐Helmholtz Platz 1 76344 Eggenstein‐Leopoldshafen Germany; ^4^ Glassomer GmbH In den Kirchenmatten 54 79110 Freiburg Germany; ^5^ Freiburg Materials Research Center (FMF) University of Freiburg 79401 Freiburg im Breisgau Germany; ^6^ Freiburg Center of Interactive Materials and Bioinspired Technologies (FIT) University of Freiburg D‐79110 Freiburg Germany

**Keywords:** additive manufacturing, amorphous metal, fused silica glass, injection molding, metal casting, tooling, two‐photon lithography

## Abstract

The growing importance of submicrometer‐structured surfaces across a variety of different fields has driven progress in light manipulation, color diversity, water‐repellency, and functional enhancements. To enable mass production, processes like hot‐embossing (HE), roll‐to‐roll replication (R2R), and injection molding (IM) are essential due to their precision and material flexibility. However, these processes are tool‐based manufacturing (TBM) techniques requiring metal molds, which are time‐consuming and expensive to manufacture, as they mostly rely on galvanoforming using templates made via precision microlithography or two‐photon‐polymerization (2PP). In this work, a novel approach is demonstrated to replicate amorphous metals from fused silica glass, derived from additive manufacturing and structured using hot embossing and casting, enabling the fabrication of metal insets with features in the range of 300 nm and a surface roughness of below 10 nm. By partially crystallizing the amorphous metal, during the replication process, the insets gain a high hardness of up to 800 HV. The metal molds are successfully used in polymer injection molding using different polymers including polystyrene (PS) and polyethylene (PE) as well as glass nanocomposites. This work is of significant importance to the field as it provides a production method for the increasing demand for sub‐micron‐structured tooling in the area of polymer replication while substantially reducing their cost of production.

## Graphical abstract

Photochemically, curable silica nanocomposites are employed to replicate master structures created through two‐photon polymerization in the sub‐micrometer regime. The resulting fused silica glass replication exhibits high‐temperature stability, serving as a template for the metal casting and hot embossing of amorphous metals. This process enables the rapid and cost‐efficient production of sub‐micron‐structured tooling.

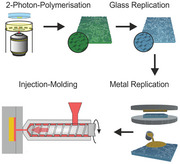

## 1 Introduction

Sub‐micrometer structured surfaces are of great importance for a wide variety of applications from optical elements for precise light manipulation, and structural colors without the need for dyes, to super‐repellent, antibacterial, or non‐reflective surfaces benefiting sensor technology and optical systems.^[^ ^1^, ^2^, ^3^, ^4^, ^5^ ^]^ Especially in the field of optics and photonics, diffractive optical elements (DOEs) featuring sub‐micrometer scale structures have gained substantial importance, particularly in the fields of telecommunication, laser processing, imaging, 3D sensing, and virtual reality.^[^ ^2^, ^3^, ^4^ ^]^ DOEs allow for the production of compact optical systems, reducing material usage and component size compared to traditional lens‐based setups. This development is critical in miniaturizing optical systems from consumer electronics and the automotive industry all the way to minimally invasive surgery where high‐resolution components are required, e.g., for endoscopes.^[^ ^6^ ^]^

For the large‐scale production of such structures in polymers, only a limited set of processes can be considered feasible including hot embossing (HE), roll‐to‐roll replication (R2R), or injection molding (IM).^[^ ^6^, ^7^ ^]^ However, these methods are all tool‐based manufacturing (TBM)‐techniques requiring the fabrication of a metal tools containing the inverse structure. The fabrication of these sub‐micrometer structured molds however remains challenging.^[^ ^8^, ^9^ ^]^ Although advancements have been made in ultra‐precision machining, such as diamond turning, for optical structures in the millimeter to micrometer range, sub‐micrometer structures are, so far, impossible to manufacture.^[^ ^10^, ^11^ ^]^ For the production of structures with resolution in the sub‐micrometer range, electroplating of lithographically or additively fabricated templates is commonly used to fabricate metallic insets mostly in nickel (a process often referred to as galvanoforming).^[^ ^12^ ^]^ Unfortunately, this is a slow method with growth rates of only a few micrometers per hour and thus tooling costs for components can easily scale way beyond economic feasibility. Furthermore, the process presents unique challenges that make it difficult for general use, including uniformity in replication, edge artifacts, proper material selection, maintaining precise process control, and post‐treatment steps, such as removing the master structure without damaging the replica.^[^ ^13^, ^14^ ^]^ Depending on the specific geometry, tool insets alone can quickly cost several thousand to tens of thousands of euros. Amorphous metals have been described as a promising material system for making similar tools. Micro hot embossing of direct reactive ion etched (DRIE) structured silicon substrates has been shown to be suitable for shaping amorphous metals to suitable tools.^[^ ^14^ ^]^ However, after the molding process, the silicon substrate is removed by etching and is thus single‐use only.^[^ ^14^, ^15^ ^]^ Furthermore, DRIE can be a complex and time‐consuming process, especially when etching deep and high aspect structures, resulting in long fabrication times. The precise control of the resulting etch depth and structure uniformity can be technically challenging requiring specialized equipment and expertise.^[^ ^16^ ^]^

We have previously introduced a novel method based on metal casting from additively derived fused silica glass templates, which for the first time enabled rapid fabrication of tool insets with single‐micron resolution.^[^ ^16^ ^]^ In this process, we cast metals such as bronze, brass, and cobalt‐chromium onto microstructured fused silica glass templates, which had been shaped using silica nanocomposites and subsequently debinded and sintered from templates fabricated using two‐photon‐polymerisation (2PP).^[^ ^17^ ^]^ Although the process was able to fabricate microstructures, it was incapable of fabricating sub‐micron features. This shortcoming is due to two reasons: First, the utilized glass replication process was, so far, only capable of reproducing features down to a few microns.^[^ ^18^, ^19^ ^]^ Second, the metals used, show large grain structures of up to 500 µm with grain boundaries and preferred grain growth directions, hindering higher resolution during metal casting.^[^ ^20^ ^]^ In the work at hand, we have tailored the glass replication process to allow, for the first time, the replication of sub‐micron features in fused silica glass parts with a resolution down to 300 nm and a surface roughness of only 3 nm. Instead of conventional metals, we used amorphous metals shaped from the fused silica nanocomposites via casting and hot embossing, allowing the accurate and smooth replication of sub‐micron features in metal. The processed, partially crystallized metal replications show high hardness and could be used as tools for polymer injection molding, replicating over 300 polymer parts with no signs of wear.

## 2 Process of High‐Resolution Tool Replication

**Figure 1:**
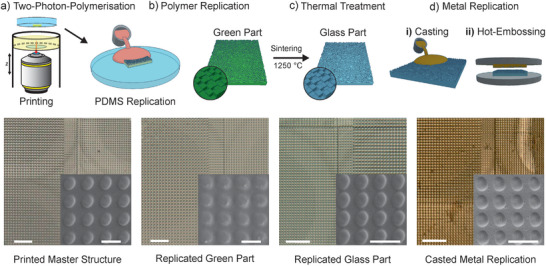
Process from printed polymer master structure to metal replica. a) The master (positive) structure is fabricated using two‐photon‐polymerization from photopolymer, which is subsequently copied into PDMS via casting (negative) (scale bar: 50 µm, inset scale bar: 5 µm). b) Replication of the PDMS master using photocurable silica nanocomposites (scale bar: 50 µm, inset scale bar: 5 µm). c) Heat treatment of the cured green part using thermal debinding and sintering to a fully‐dense and transparent fused silica glass replication (positive) (scale bar: 50 µm, inset scale bar: 5 µm). d) By casting (i) or hot embossing (ii) of an ZrNi based alloy, capable of forming metallic glass, against the sintered fused silica glass part (negative) (scale bar: 50 µm, inset scale bar: 5 µm).

**Figure** **1** illustrates the workflow of the method developed in this work. The master structures were printed by 2PP using the commercial system NanoOne (UpNano GmbH) (Figure 1a). These templates were subsequently transferred into polydimethylsiloxane (PDMS) (Figure 1b). The PDMS replicates were then transferred into highly temperature‐stable fused silica glass using a silica nanocomposite approach.^[^ ^18^, ^19^ ^]^ The silica nanocomposite is hereby poured onto the PDMS template and cured using UV light resulting in the so‐called green part. The polymerized part is then thermally debinded at 600 °C and sintered to full density at 1250 °C (Figure 1c). In the final step, the fused silica glass structure is transferred into amorphous metal using casting or hot embossing. The newly developed possibility of printing fused silica glass directly via 2PP would also make it suitable for printing a metal casting template, but since 2PP is a time‐consuming process, especially for printing larger specimens, it was decided to use the soft replication method to save processing time.^[^ ^17^ ^]^

Previous reports used silica nanocomposites with a silica particle size in the range of ≈50–100 nm, which are not capable of replicating structures in the sub‐micrometer regime. In general, it can be noted that the smaller the particles in the nanocomposite, the higher the replication fidelity. In order to enable the precise replication of fused silica glass templates with sub‐micron resolution, we used nanocomposites consisting of silica nanoparticles with a diameter of ≈10 nm. This allowed, for the first time, the replication of sub‐micron scale structures with minimum features down to 300 nm using the silica nanocomposite approach. The use of small particles enables a lower sintering point, which was determined using dilatometer measurements (see Figure S1, Supporting Information). The surface roughness of the sintered glass samples was measured to be 3 nm (S_q_) using white light interferometry (WLI) on a surface area of 40 × 40 µm^2^. The silica nanocomposites used in this work had a solid loading of 24 vol%, resulting in an isotropic linear shrinkage of 37.9 %. This shrinkage is in good accordance with the calculated linear shrinkage of 37.8 % (see suppoTable S1, supporting information).

For the replication in metal, two processes were investigated: metal casting and hot embossing. Since the glass components produced are made of fused silica glass, they are extremely temperature shock resistant, and can withstand high temperatures of up to 1600 °C and rapid temperature changes. In this work, we used a zirconium‐based metal alloy, capable of forming metallic glass, for casting and hot embossing. The casting process is shown schematically in Figure 1d,i). The metal is molten in an inductive furnace at a temperature of 1300 °C and cast under vacuum onto an embedded fused silica glass replication. Afterward, an overpressure of argon is applied to ensure sufficient mold filling. The glass can be removed by hand when the metal has fully solidified without the need for chemicals or mechanical force. The second investigated processing method (shown in Figure 1dii) is hot embossing, exploiting the softening point of the amorphous material at ≈450 °C (see Figure S1, supporting information). In this state, the material can be hot embossed with the glass part serving as the embossing die. The transfer of the structures was conducted using a hot embossing system under air, requiring the metal to be coated with gold preventing the metal from oxidation during hot embossing.

## 3 Replication Quality and Characterization

**Figure 2:**
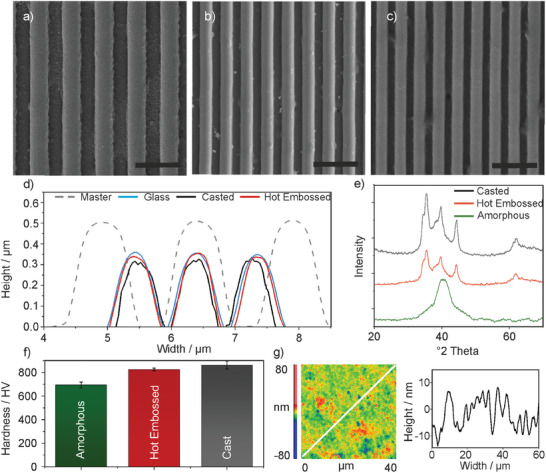
Characterization of the fused silica and metal replications. a) Representation of a master with a feature size of 820 nm. (scale bar: 2 µm). b) Representation of the replicated fused silica component. During the sintering process the part shrinks to a structure size of 510 nm (scale bar: 2 µm). c) SEM of the replicated metal component casted from the fused silica glass structure, with a structure size of 500 nm. (Scale bar: 2 µm). d) AFM measurement of the produced structures in metal via casting (black) and hot embossing (red) to determine the achievable casting accuracy in comparison with the master (gray dashed) and the fused silica glass replica (blue). e) XRD pattern of the amorphous metal prior to the replication (green), after hot embossing (red), and after casting (black). f) Vickers hardness measurement of the amorphous metal prior to the replication process (green), after and hot embossing (red) and casting (black). g) Measurement of the surface roughness of a metal component after casting, with a surface roughness of Sq ≈9 nm.

In order to demonstrate the quality of structure replication, a simple lines‐and‐spaces pattern was investigated. **Figure** **2**a shows the master structure with a structure width of 820 nm. The replications produced in fused silica glass generate the line pattern with a structure width of ≈510 nm (see Figures 2b,c) shows the replication in the metal of the same structure, with a final structure width of ≈500 nm (The structures were measured on the straight flanks at 50 % of their respective heights). In order to determine the replication quality, the master structure, the replicated glass structure, and the cast as well as hot embossed metal structures were measured using Atomic Force Microscopy (AFM, see Figure 2d). The measurements revealed the successful replication of the master structure, validating the expected shrinkage. A comparison between the casted and hot‐embossed metal samples, both featuring a structural size of 500 nm, highlights that hot embossing exhibits superior replication accuracy (see Figure 2d). The better replication quality of the structures shown in Figure 2d using hot embossing is explained by the circumstance that on the one hand only thermal shrinkage but no solidification shrinkage takes place, and on the other hand a constant pressure and increased temperature is maintained during the hot embossing process, which gives the material time to adapt to the glass mold. The aspect ratio that can be derived from the structure shown in Figure 2d is ≈0.4.

X‐ray diffraction (XRD) analysis of the amorphous raw material, as well as the cast (at 1300 °C) and hot embossed (at 450 °C) samples, were carried out to investigate the material structure after processing (see Figure 2e). Both casted and hot embossed metal samples show crystalline and amorphous regions, indicating an expected partial crystallization of the parts during the process.^[^ ^21^, ^22^ ^]^ Vickers Hardness of the amorphous as well as the processed samples in this work was measured as it has a direct influence on the fatigue strength and thereby has a direct impact on the service life of the manufactured tool.^[^ ^8^, ^23^ ^]^ Vickers hardness of the amorphous material was measured to be 695 ± 25HV, while the values after casting or hot embossing are increased to 863 ± 34HV and 826 ± 12 HV, respectively (see Figure 2f). Surface roughness of the metal replications was measured using WLI showing a roughness of 9.0 ± 0.3 nm (S_q_) for the cast and 7.0 ± 0.2 nm (S_q_) for the hot embossed samples on an area of 40 × 40 µm^2^ (see Figure 2g). The measurements show that the surface roughness in casted samples is higher than in metal parts produced by hot embossing (see Figure S3, supporting information).

**Figure 3:**
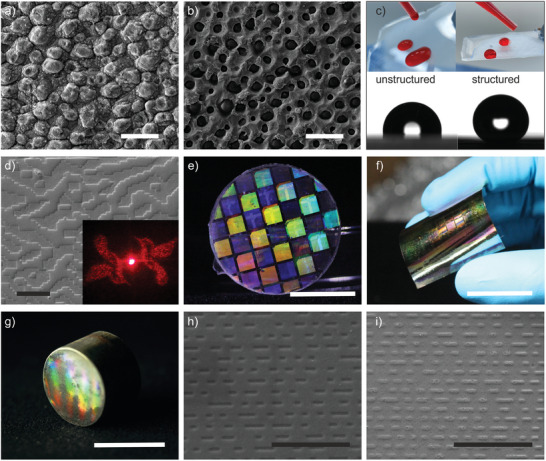
Various examples from nature and technology demonstrating the wide applicability of the described sub‐micron replication method. a) SEM image of a leaf of the Nelumbo nucifera Lotus surface replicated in fused silica glass (positive) (scale bar: 25 µm). b) SEM image of the leaf surface replicated in casted amorphous metal (negative) (scale bar: 25 µm). c) Comparison of the beading effect on a structured and unstructured surface as well as the contact angle on a structured and unstructured surface replicated in epoxy from the casted metal part. d) SEM image of the surface of a replicated DOE structure in metal (scale bar: 20 µm), the inset shows the projection of the DOE. e) Illustration of a replicated fused silica part with a color effect produced by a microstructure on the surface (scale bar: 1 cm). f) Illustration of the amorphous metal foil manufactured by hot embossing (scale bar: 2 cm). g) Replicated metal inset of a CD‐ROM (scale bar: 2 cm). h) SEM image of the CD structure on a replicated fused silica part (scale bar: 5 µm). i) SEM image of the CD structure in hot embossed metal (scale bar: 5 µm).

A variety of sub‐micron structures were fabricated to demonstrate the diversity of applications that can be addressed using the demonstrated process. A lotus leaf was replicated into metal and copied into epoxy resins to demonstrate the fabrication of super‐repellent surfaces. **Figure** **3**a shows an SEM image of the replicated fused silica glass surface, Figure 3b the corresponding metal casting replication. Figure 3c shows the water‐repellent properties as well as the contact angle with and without structuring on commercial epoxy resin, replicated from the casted metal structure. An increase of the static contact angle from 100° to 150° compared to the non‐structured polymer part was determined. An exemplary DOE was designed, 3D‐Printed, and replicated to demonstrate the applicability to fabricate micro‐optical elements in metal (see Figure 3d). The DOE was designed to project the images of two birds when illuminated with a laser. To investigate the difference in quality between the glass and metal replication, the structures were compared using a light microscope and their corresponding generated holograms (GH) were visually compared (see Figure S4, supporting Information). To demonstrate structural coloration a variety of structures with features between 500 nm and 10 µm were replicated (see Figure 3e) showing different structural coloration depending on the viewing angle. This type of structural coloration is caused by diffraction, incoming light is scattered, if the wavelength of the light is comparable to the dimensions of the light.^[^ ^24^ ^]^ The same structures were also used to structure thin amorphous metal foils, demonstrating that these foils can be imprinted and remain flexible (see Figure 3f). The smallest structure replicated in this work originates from a CD‐ROM. After the replication step to metal, it features a structure width of below 300 nm (see Figure 3 g–i). The produced metal inset is depicted in Figure 3g, exhibiting a color effect on the surface.Figure 3h,i display the SEM measurements of the produced fused silica glass and metal replication, providing a detailed view of the structure. This structure could also be transferred to metal foils by hot embossing and measures 60 nm in height (see Figure S6, supporting information).

## 4 Injection Molding using Replicated Metal Insets

**Figure 4:**
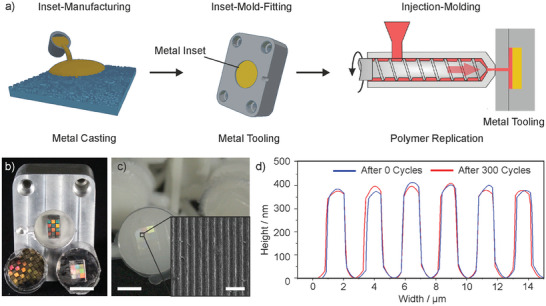
Injection molding using cast metal insets. a) Schematic representation of the production process and the application of the manufactured metal insets in the injection molding process. b) The metal insets used in injection molding, produced via casting (scale bar: 10 mm). c) Injection molded polymer parts (PE) using the metal mold from b (scale bar: 10 mm, inset scale bar: 50 µm). d) WLI of the casted metal structure before (blue) and after (red) 300 injection molding cycles.

The metal insets were assembled in an injection molding inset tool made of aluminum and tested by replicating various thermoplastic polymers including polyethylene (PE) and polystyrene (PS) (**Figure** **4**). Furthermore, we tested thermoplastic silica nanocomposite with up to 50 vol% silica solid loading. The two different polymers were tested, to assess the behavior of the injection mold at different temperatures of 140 and 260 °C, respectively (see Figure 4c). In order to obtain data on the durability of the prepared metal insets, over 300 replications were carried out. Before and after use, the inset was analyzed using WLI to measure its surface structure and detect any deformation or signs of wear. As can be seen, the metal surface and structures show no signs of wear after 300 cycles (see Figure 4d). Smaller observable differences result from variations in measurement points before and after the metal inset was used in injection molding.

In this work, we have demonstrated a novel manufacturing process that enables the replication of metal insets with sub‐micron resolution and low surface roughness, based on two‐photon‐polymerization, fused silica replication, metal casting, and hot embossing. We demonstrated the structuring of fused silica glass as well as amorphous metal alloys based on zirconium and nickel with feature sizes below 300 nm and a surface roughness below 10 nm (S_q_). The resulting metal replicas have been used in polymer injection molding showing no wear after 300 cycles. This process thus enables, for the first time, the flexible production of metal insets from fused silica glass with structures in the range of less than one micrometer by means of a casting or hot embossing process.

## 5 Experimental Section

### Materials

The 2‐photon resin UpBrix was provided by UpNano (Austria). Glassomer µL24 as well as the Glassomer Hardener were provided by Glassomer (Germany). The PDMS, Elastosil M4601, was purchased from Wacker (Germany). The Plaster “Pro‐HT Platinum” was purchased from Horbach Technik (Germany). The amorphous metal alloy Amloy‐Zr02 was provided by Heraeus (Germany). The amorphous metal foil Vitrobraze VZ2152 was provided by VAC (Germany). For replication of the lotus leaf from the manufactured metal replication, the commercial epoxy resin E45 was purchased from BEKATEW GmbH & Co.KG (Germany)

### Two‐Photon‐Polymerization

The 2PP master structures were printed using the high‐resolution printing system “NanoOne” printing system from UpNano GmbH (Austria) in Vat mode equipped with a 60x oil immersion objective (NA 1.42, UPLXAPO60XO, Olympus). The laser (80 MHz repetition rate, 90 fs pulse length, 780 nm center wavelength) was focused through a glass cover into a resin vat containing the index‐matched resin UpBrix (UpNano, Austria). The structures were printed on a fused silica glass substrate with the two‐photon resin “UpBrix”. The prints were carried out using 60x magnification, at a laser power of 5 mW, a printing scanning speed of 50 mm^−1^s, a line distance of 150 nm, and a layer height of 100 nm in fine infill mode.

### Replication of the Master Structures

PDMS was mixed in a container for 2 min in a ratio of 9:1 by weight (A:B component). To remove air bubbles from the mixture, a desiccator was used. The structure to replicate was fixed in a Petri dish and then replicated using PDMS, which was cured in an oven at 60 °C for 1 h. The cured PDMS replica was removed carefully from the master structure. Glassomer µL24 was mixed with Glassomer Hardener according to the manufacturer's specifications. Glassomer µL24 was then applied onto the replicated PDMS template and cured by UV illumination at a wavelength of 320–405 nm for 2 min. After curing, the nanocomposite was removed from the PDMS mold.

### Heat Treatment

To remove the polymeric component of the nanocomposite, thermal debinding of the cured Glassomer green parts was done using an ashing furnace (AAF, Carbolite Gero, Germany) at 600 °C. The resulting brown parts could be sintered in a tube furnace (STF16/450, Carbolite/Gero, Germany) at 1250 °C and a vacuum of 5 × 10^−3^ mbar.

### Fused Silica Embedding

To stabilize the replicated fused silica components during the metal casting, the fused silica glass templates were fixed in a steel cuvette using the high‐temperature embedding material (Pro‐HT Platinum, Horbach Technik, Germany). The high‐temperature embedding material was prepared by the manufacturer's specifications, mixing the powder with water in a ratio of 31:100 by weight. The embedding material was mixed until no dry powder was left and the mixture had a homogeneous consistency and poured into the prepared metal cuvette. The metal cuvette was left to dry overnight, before heating it to 800 °C for 2 h.

### Metal Casting

In order to create the metal replication, the prepared steel cuvette with the fused silica replication was preheated to 300 °C before casting. The preheated setup was then placed in the casting furnace (M20, Indutherm, Germany). The casting camber was then flooded with argon, followed by the application of a vacuum. The material was melted at a temperature of 1300 °C under vacuum. The casting chamber was then tilted, so the molten metal could flow into the steel cuvette and onto the fused silica template. When the molten metal was fully transferred to the cuvette, a pressure of 3 bar of argon was generated in the chamber. The casting furnace was left in this position until the metal part was solidified.

### Hot‐Embossing

For the metal hot embossing the amorphous metal samples were coated with a gold layer using sputtering. The metal sample was placed on a piece of aluminum foil, on top of the metal sample the fused silica part was placed with the structured side facing the amorphous metal part. The prepared sample stack was placed in an, IMT‐modified, hot embossing machine based on a Jenoptik HEX03 / Zwick tensile testing machine. The embossing chucks were closed and heated to a temperature of 450 °C before closing fully and applying a force of 25 kN. The chucks were allowed to cool down to 150 °C before opening.

### Replication from the Metal Structures

The epoxy was mixed under stirring for 2 min in a ratio 2:1 by weight (A:B component). To remove entrapped air, the used beaker with the mixed epoxy resin was placed in a desiccator. The manufactured metal structure was replicated using the epoxy, which was cured in the oven at 60 °C for 24 h.

### Characterization

XRD measurements were carried out in Bragg‐Brentano geometry with a diffractometer (D8 DISCOVER, Bruker, Germany) with a Cu‐Kα radiation source and a LYNXEYE XE‐T detector. The roughness was measured using the WLI (NewView 9000, Zygo, USA) on an area of 40 × 40 µm^2^ (see Table S2, supporting information). The surface roughness measurements were carried out three times at different locations, the measurements were evaluated using Gwyddion. To determine the replication capability, the cross‐sections of different stages of the process (master, fused silica, metal) were measured using the AFM (Multimode 8, Bruker, Germany) on an area of 10 × 10 µm^2^. Vickers hardness was measured using the micro indentation tester (MHT‐10, Anton Paar, Germany) with a load of 1000 N and a load duration of 1 s. The mean value was determined from three individual measurements. To determine the contact angle of the structured surfaces, the contact angle measuring device (OCA 15Pro, Data Physics, Germany) was used. For the measurement of static contact angles, 5 µl water droplets at room temperature were used according to the sessile droplet method. To generate a valid measurement, three contact angles were measured at different positions on the sample and these values were used to calculate the average.

## Conflict of Interest

The Glassomer GmbH has patented the technology described within this paper (application/patent no. EP20195971.5) and is in the process of commercializing it. The authors declare no other competing interests.

## Supporting information

Supporting Information

## Data Availability

The data that support the findings of this study are available in the supplementary material of this article.
