# Supplementary material for: Sub‐Micron Replication of Fused Silica Glass and Amorphous Metals for Tool‐Based Manufacturing
Source: Adv Sci (Weinh). 2024 Jul 12;11(35):2405320. doi: 10.1002/advs.202405320 (PMC11425202; doi:10.1002/advs.202405320)

Supporting Information

**Sub-Micron Replication of Fused Silica Glass and Amorphous Metals for Tool-based Manufacturing**

*Sebastian Kluck, Richard Prediger, Leonhard Hambitzer, Manuel Luitz, Niloofar Nekoonam, Franziska Dreher, Markus Lunzer, Matthias Worgull, Marc Schneider, Bastian E. Rapp, Frederik Kotz-Helmer^*^*


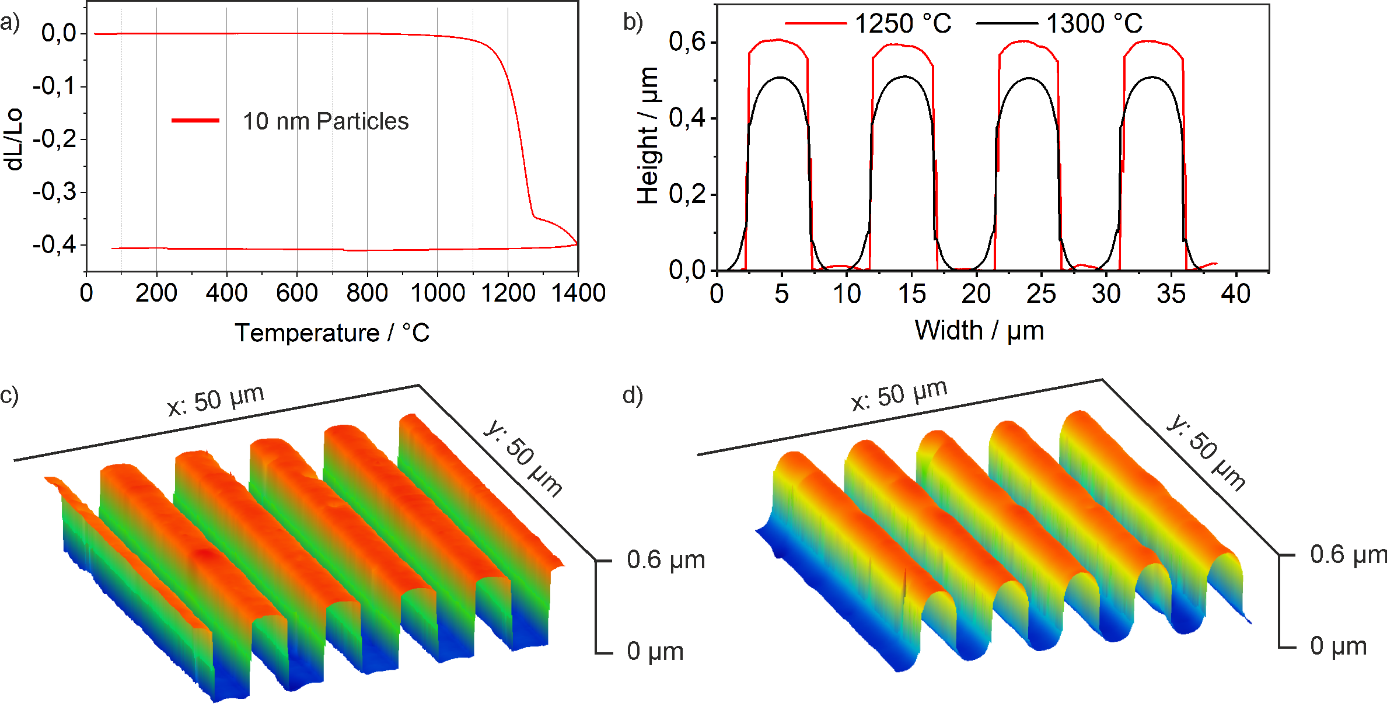


**Figure S1.** Measurements to determine the sintering properties. a) Dilatometer measurement, to determine the sintering temperature of the powder with an average particle size of 10 nm. b) WLI measurements of structures sintered at 1250 °C (red) and 1300 °C (black). c) 3D representation of the measurement from the measured structure sintered at 1250 °C. d) 3D representation of the measurement from the measured structure sintered at 1300 °C

The dilatometer measurement depicted in Figure S1 was conducted to determine the sintering temperature of the silica powder, which has a particle size of approximately 10 nm. This is crucial to avoid setting the sintering temperature too high, which could potentially damage surface microstructures. As shown in Figure S1, the exact sintering temperature is important to not compromise the structural integrity of micro- or submicro-structured glass samples. Figure S1b compares samples which were identically manufactured but sintered at different temperatures. The WLI measurements show that the edges of the structures significantly contract and lose approx. 100 nm of their height. Figure S1c, d shows the 3D measurements of the differently sintered structures, it can be observed that this effect occurs on all edges on both the top and bottom of the structures. Therefore the correct temperature setting is crucial.

In order to calculate the theoretical shrinkage Y_s_ equation (1) is used, which depends on the solid loading Φ, the theoretical density ρ_t_ and the final density ρ_f_, of the produced part. The actual shrinkage was determined by measuring the dimensions of the produced parts in the green state and in sintered state by analyzing SEM measurements with the image analysis program Image J.

$\boldsymbol{Y}_{\boldsymbol{s}}\boldsymbol{=1-}\left( \frac{\boldsymbol{\Phi}}{\frac{\boldsymbol{\rho}_{\boldsymbol{t}}}{\boldsymbol{\rho}_{\boldsymbol{f}}}} \right)^{\frac{\boldsymbol{1}}{\boldsymbol{3}}}$ (1)

In order to determine the shrinkage of the material system, the width and height of the structures were measured as a polymer component and as a finished sintered dense glass component, the results are listed in Table S1. The shrinkage is 37.9 % laterally and 38.0 % vertically and is therefore close to the theoretical shrinkage of 37.8 %.

| No. | Green part | | Sintered part | | Linear shrinkage | |
| --- | --- | --- | --- | --- | --- | --- |
|  | Width / µm | Height / µm | Width / µm | Height / µm | *Ys Width* / % | *Ys Height* / % |
| 1 | 135.9 | 0.899 | 84.9 | 0.555 | 37.5 | 38.3 |
| 2 | 135.6 | 0.894 | 84.2 | 0.561 | 37.9 | 37.2 |
| 3 | 135.6 | 0.890 | 84.2 | 0.557 | 37.9 | 37.4 |
| 4 | 135.9 | 0.899 | 84.4 | 0.553 | 37.9 | 38.5 |
| 5 | 135.4 | 0.921 | 83.8 | 0.563 | 38.1 | 38.8 |

**Table S1.** Measurement of the shrinkage characteristics of replicated fused silica glass throughout the sintering process. Linear shrinkage (Ys) through data obtained from shrinkage measurements conducted on casted fused silica. The measured dimensions of the replicated parts was measured in two directions, both in the green part state and after sintering to full density, aiming to validate the isotropic shrinkage.

**
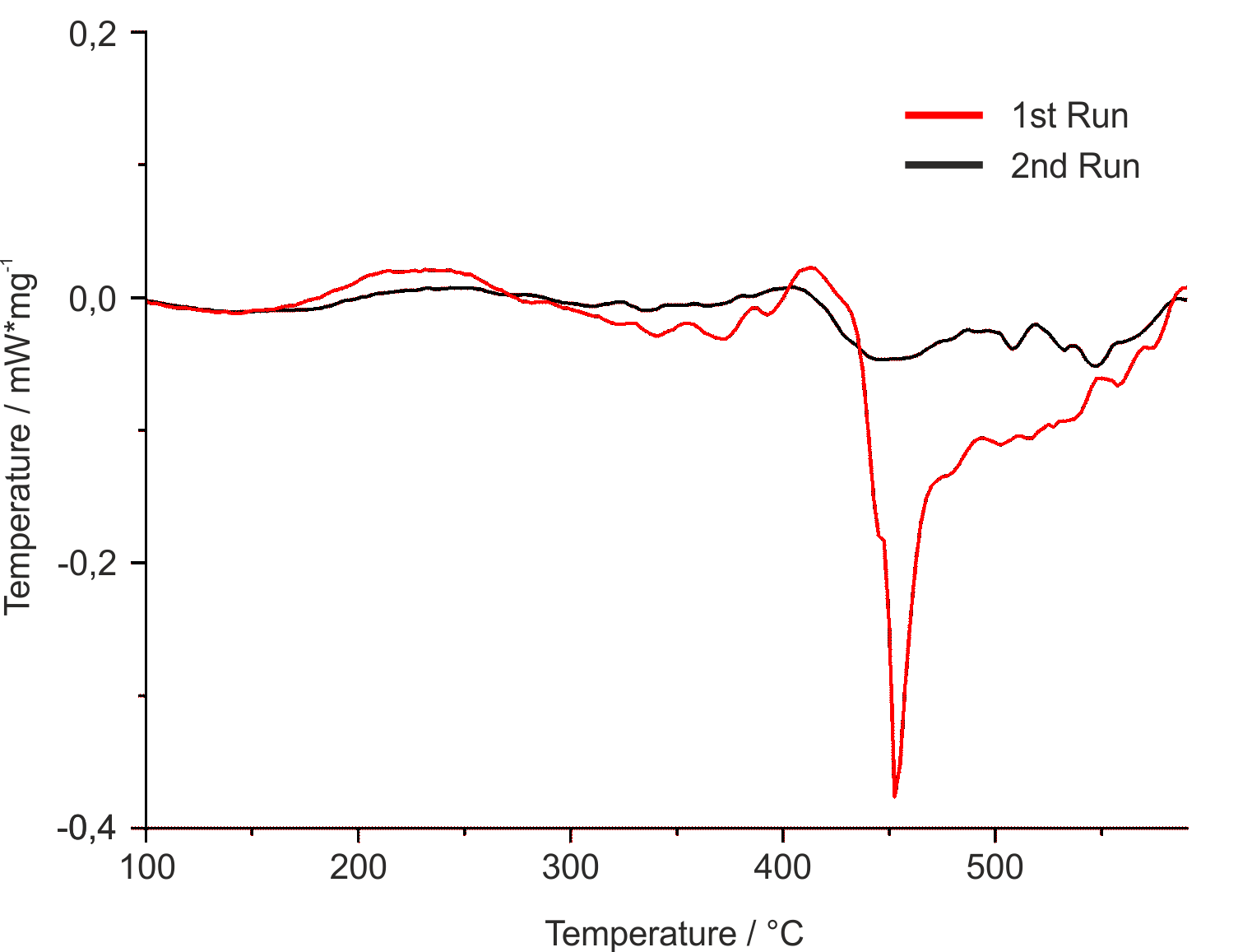
**

**Figure S2.** DSC measurement of the metal alloy used for casting and hot embossing. The first run shows the material and its glass transition point, as well as the crystallization of the material. The second run with the same sample shows no further crystallization reaction.

We further evaluated if the surface structure has an impact on the roughness of the final metal component, for this the line roughness R_q_ on casted metal structures was measured both on the top and bottom of the structures, Figure S3e shows the structure measured by white light interferometry. The measured roughness values are 6.4 nm (R_q_) and 5.9 nm (R_q_) for the top and bottom side respectively. To illustrate the uniformity of the manufactured structures, a 3D image of a 100 x 100 µm² surface was recorded, which shows that the structure is homogeneous over the entire surface (see Figure S3f).

**
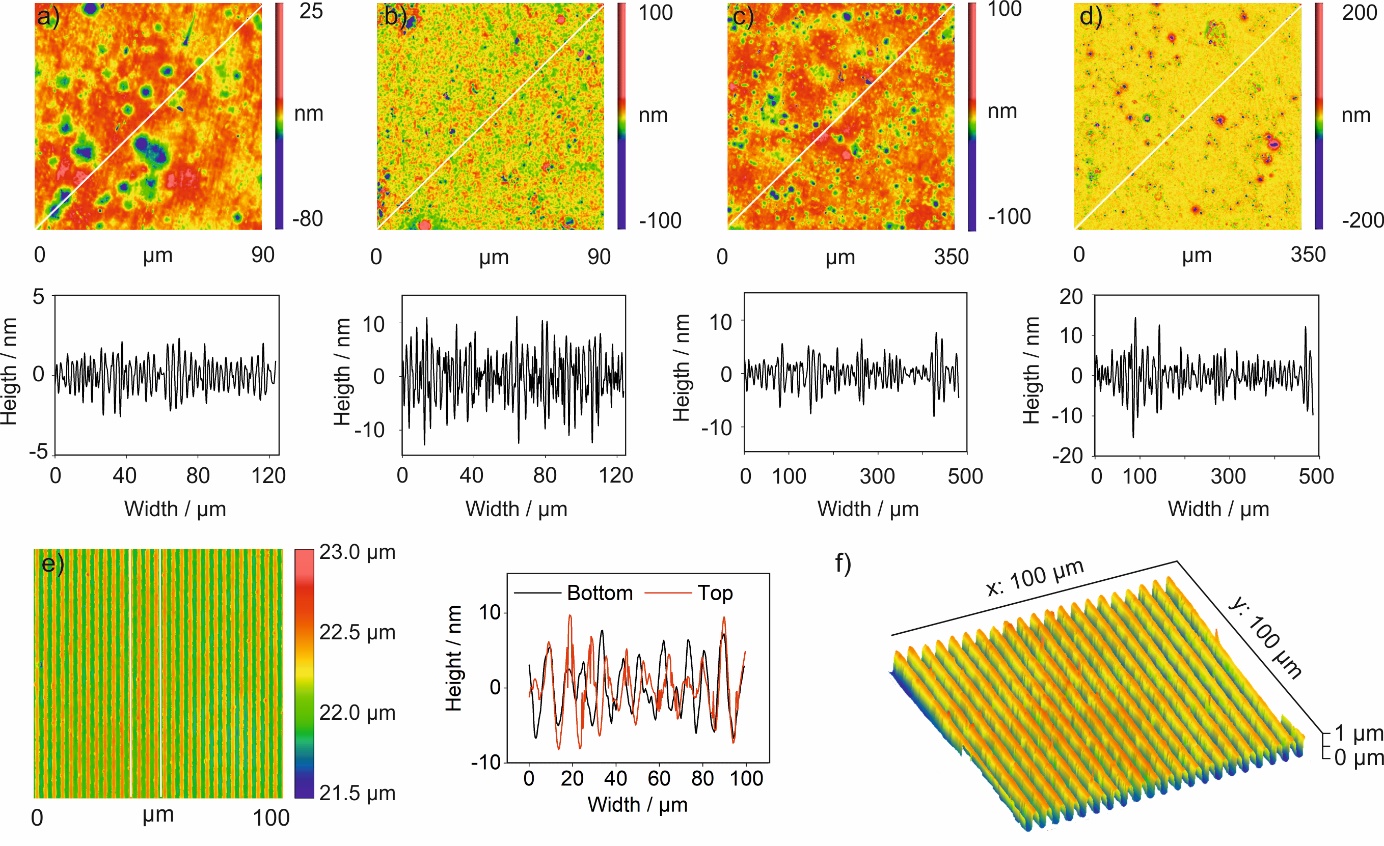
**

**Figure S3.** Measurements of the surface roughness on unstructured and structured metal replications a) WLI surface roughness measurement with an area of 90 x 90 µm^2^ on hot embossed unstructured metal with a surface roughness of 8.0 ± 0.3 nm (S_q_). b) WLI surface roughness measurement with an area of 90 x 90 µm^2^ on casted unstructured metal with a surface roughness of 16.7 ± 1.0 nm (S_q_). c) WLI surface roughness measurement with an area of 350 x 350 µm^2^ on hot embossed unstructured metal with a surface roughness of 14.3 ± 1.7 nm (S_q_). d) WLI surface roughness measurement with an area of 350 x 350 µm^2^ on casted unstructured metal with a surface roughness of 29.5 ± 0.8 nm (S_q_). e) WLI surface roughness measurement with an area of 100 x 100 µm^2^ on casted structured metal the line roughness was measured to be 6.4 ± 0.1 nm (R_q_) on the top of the structure and 5.9 ± 0.2 nm (R_q_) on the bottom of the structure. f) 3-Dimensional image of the metal structure investigated to show the homogeneity over the entire measuring area.

In order to validate the quality of the manufactured components, further roughness measurements were carried out on a larger surface. The measurements in Figure S3 show roughness measurements on unstructured components with a measuring area of 90 x 90 µm² (see Figure S3a, b) and 350 x 350 µm² (see Figure S3c, d), in each case for hot embossed and casted samples. The roughness increases with increasing surface area and is lower for the hot embossed samples than for the cast components. This can be attributed on the one hand to the investment material used for casting, which can be a source of dust causing surface defects, and on the other hand to turbulence in the melt during casting. These sources of defects are avoided with hot embossing, and the sample surface is smoothed during hot embossing by pressing on the smooth glass components. The roughness values for glass, casted and hot embossed metal are given in Table S2.

| Area | Replicated Glass | Casted metal | Hot embossed metal |
| --- | --- | --- | --- |
| *µm²* | *Sq / nm* | *Sq / nm* | *Sq / nm* |
| 40 x 40 | 2.9 | 9.2 | 7 |
| 40 x 40 | 2.9 | 10.1 | 7.3 |
| 40 x 40 | 2.8 | 9.8 | 7.6 |
| 90 x 90 | 5.3 | 15.4 | 7.6 |
| 90 x 90 | 5.9 | 17.6 | 8.3 |
| 90 x 90 | 4.8 | 17.2 | 8.2 |
| 350 x 350 | 7.6 | 29.5 | 16.1 |
| 350 x 350 | 9.1 | 28.5 | 12.0 |
| 350 x 350 | 7.9 | 30.4 | 14.8 |

**Table S2.** Surface roughness of the produced fused silica and metal replications. Surface roughness *S_q_* (root mean square) of the replicated fused silica and metal replications measured using WLI on an area of 40 x 40 µm^2^, 90 x 90 µm² and 350 x 350 µm².

To provide an application example of the process described here, DOE structures were printed and transferred into metal. In Figure S4, these structures were examined under a microscope, and the corresponding generated holograms (GH) were compared. As seen, the master structure exhibited high resolution in its physical form but not in the GH (see Figure S4a). This can be explained by the fact that the DOE structure was calculated for a pixel size of 10 µm. However, to achieve this size, the master structure had to be printed significantly larger (approximately 16 µm per pixel) due to material system shrinkage during the process as shown. This change in size led to inadequate resolution of the GH. As depicted in Figure S4b, the resolution of the GH in glass was significantly higher due to the reasons mentioned. Figure S4c shows the metal replication of the DOE structure, which also shows a higher resolution than the master structure, but is less clear than the glass replication.


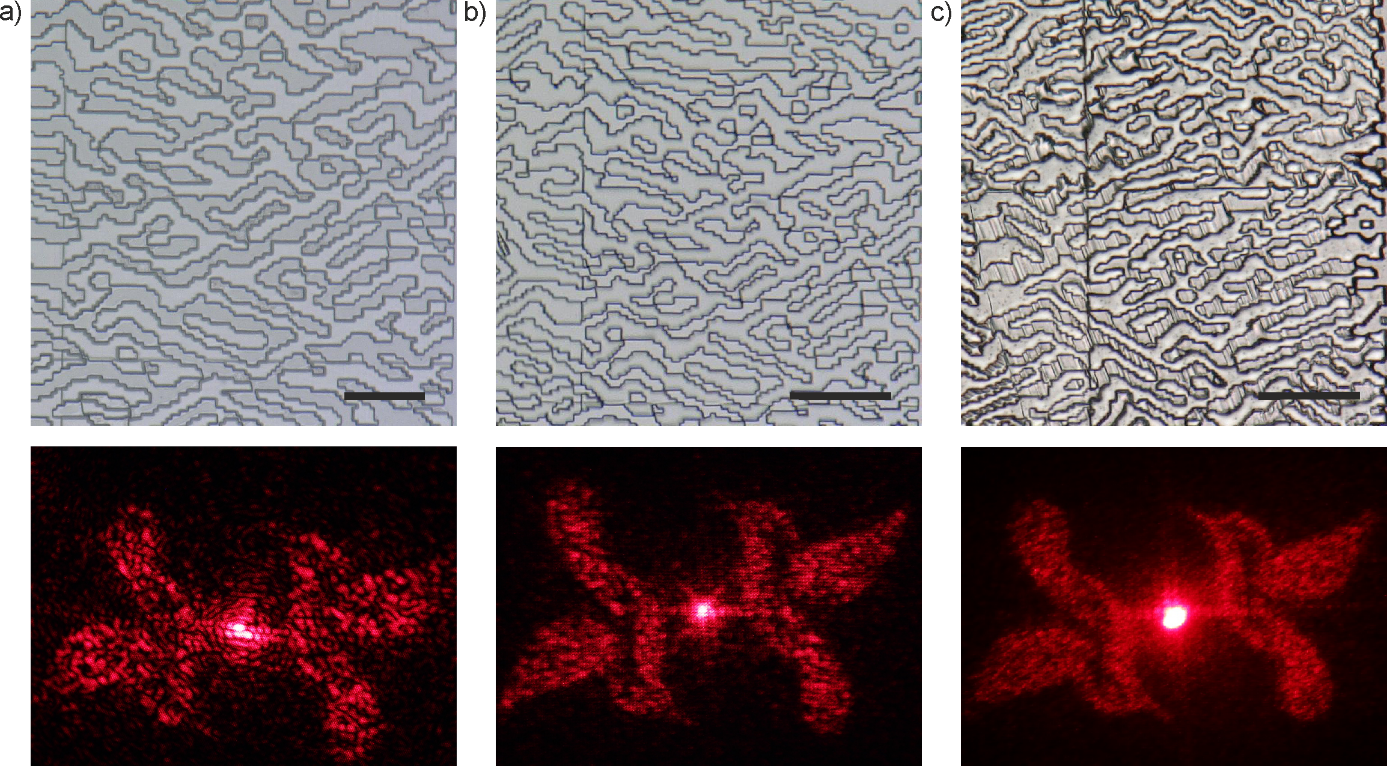


**Figure S4.** Replication of a DOE structure and its generated hologram (GH). a) The master structure (scale bar: 50 µm). b) The glass replication and its GH (scale bar: 50 µm). c) The metal replication of the DOE and its GH (scale bar: 50 µm).


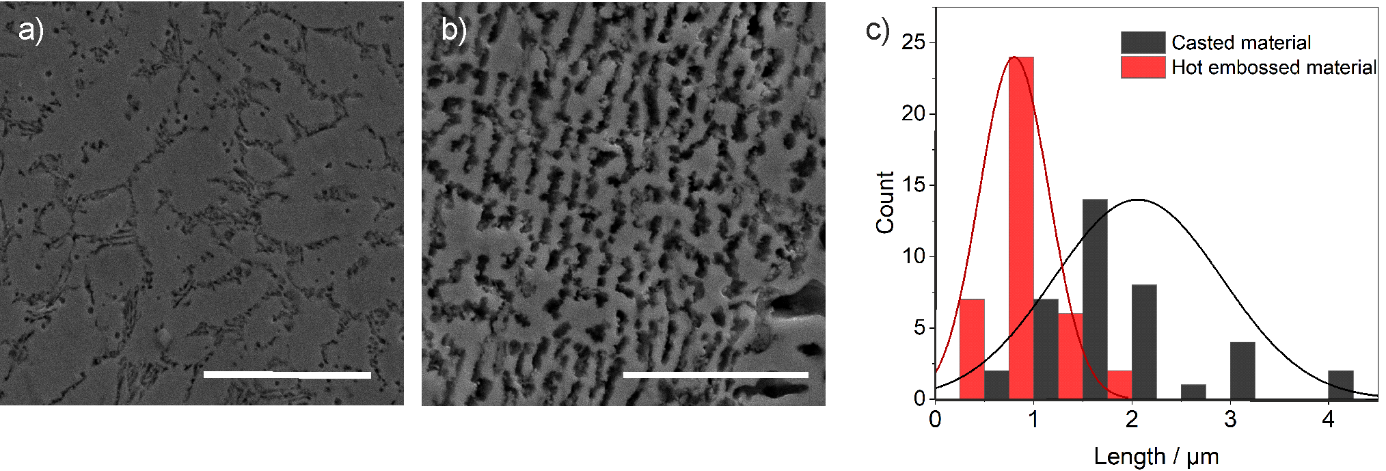


**Figure S5.** Chemical wet etching of the surface of polished metal samples produced by casting and hot embossing to measure the grain size resulting from the respective process. a) SEM image of an etched casting sample (scale bar: 5 µm). b) SEM image of an etched hot embossing sample (scale bar: 5 µm). c) Comparison of the measured grain size in casted and hot embossed samples.

Etching was carried out on casted and hot embossed samples to determine how the grain size changes as a result of the respective thermal treatment (see Figure S5). The average grain size for hot embossed and casted samples was measured to be 1.3 µm and 2.2 µm, respectively. The higher grain size for the casted samples can be attributed to the higher processing temperature of 1300 °C used in the casting process compared to hot embossing which is performed at 450 °C.

The smallest structures replicated in this work originate from a CD master. This was replicated using the process described in this work and shows structures with a width of 500 nm and a structure height of 100 nm in the master. The replicated metal show a width of 300 nm and a height of 60 nm. (The structures were measured on the straight flanks at 50 % of their respective height)


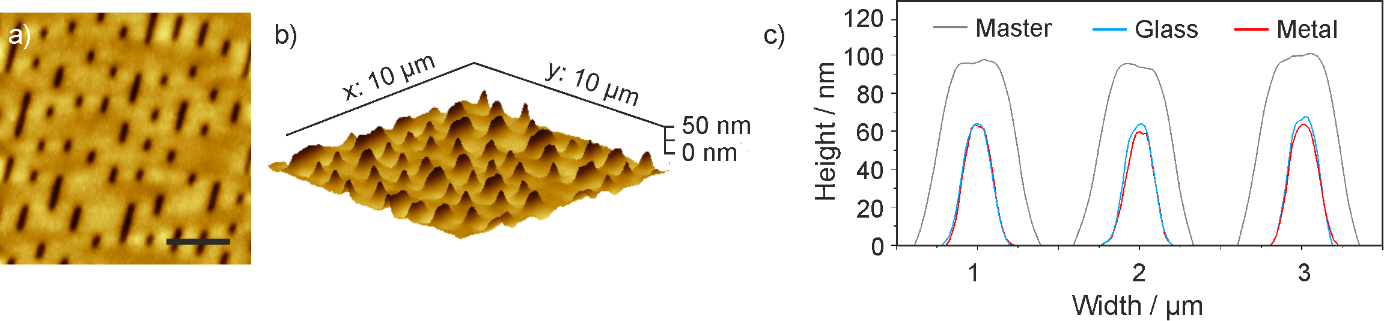


**Figure S6.** Measurement of the CD surface structure replicated in metal using AFM. a) AFM measurement with an area of 10 x 10 µm^2^ on embossed amorphous metal foil (scale bar: 2 µm). b) 3D representation of the measurement from a). c) Measurement of the structure using Gwyddion software, comparing the master structure (gray), the replicated glass template (blue) and the replicated metal structure (red).

In order to demonstrate that structures with straight walls can be replicated with this process, structures with a small draft angle of 2 ° were replicated (see Figure S7).


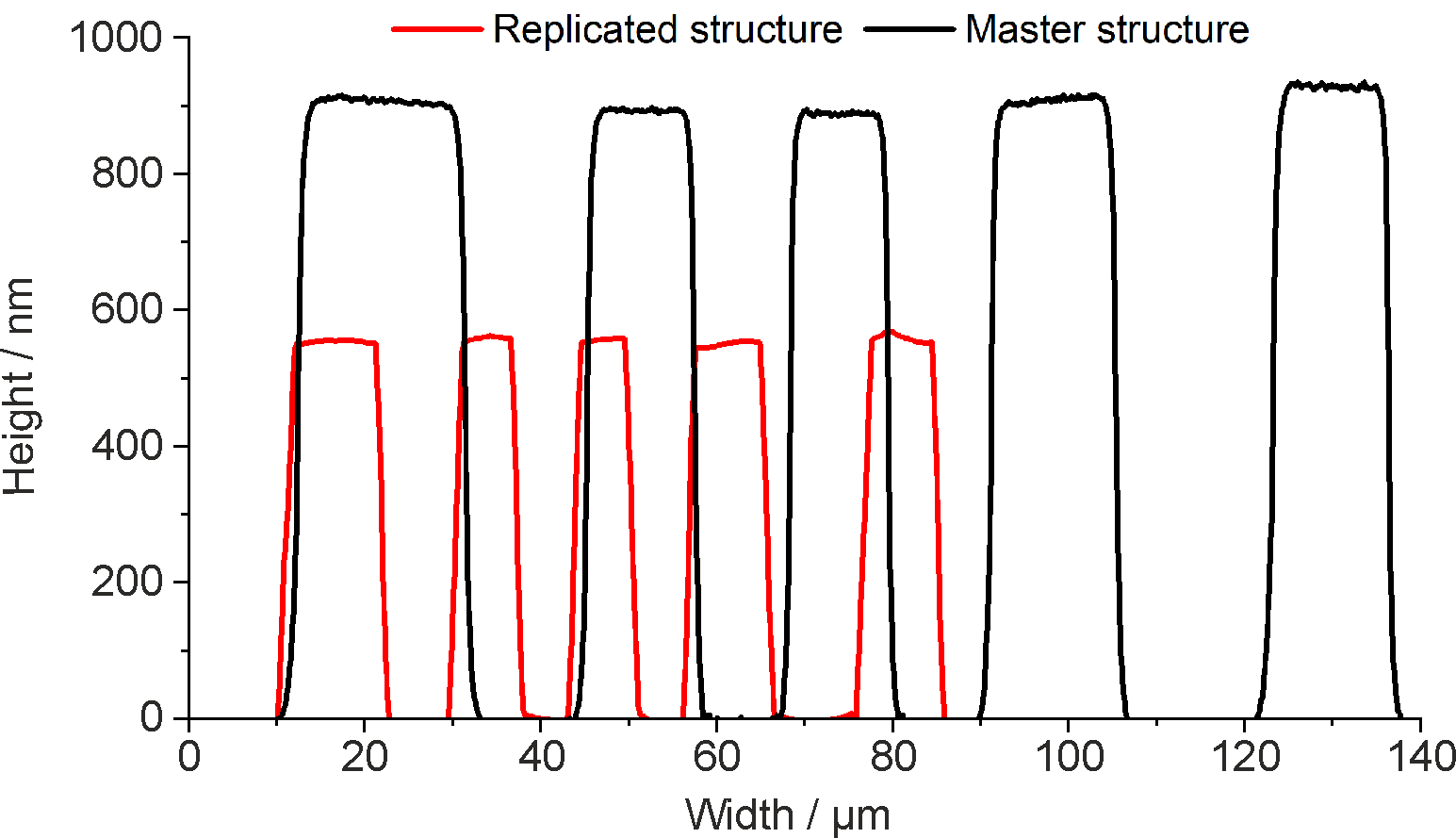


**Figure S7.** Measurement of a replicated structure as master (black) and glass (red) with straight walls and a small draft angle. The structures show an angle of 2 ° respectively to the vertical and have straight top and bottom surfaces.

| No. | Dry mass | | | Buoyancy mass | | | Calculated density | | |
| --- | --- | --- | --- | --- | --- | --- | --- | --- | --- |
|  | *m*1 / g | *m*2 / g | *m*3 / g | *m*1*_b_* / g | *m*2*_b_* / g | *m*3*_b_* / g | r1 / gcm^-3^ | r2 / gcm^-3^ | r3 / gcm^-3^ |
| 1 | 0.6831 | 0.6835 | 0.6833 | 0.3103 | 0.3107 | 0.3106 | 2.1975 | 2.1959 | 2.1960 |
| 2 | 0.6502 | 0.6504 | 0.6503 | 0.2960 | 0.2960 | 0.2962 | 2.1927 | 2.1933 | 2.1915 |
| 3 | 0.6968 | 0.6969 | 0.6967 | 0.3166 | 0.3168 | 0.3163 | 2.1969 | 2.1959 | 2.1987 |

**Table S3.** Density measurement of the casted fused silica glass system. Measurements of the dry mass and buoyancy mass of 3 different samples to calculate the density of the resulting fused silica glass parts using the Archimedes principle. The data shows that full density is achieved.

**Table of content**

Photochemically curable silica nanocomposites are employed to replicate master structures created through two-photon polymerization in the sub-micrometer regime. The resulting fused silica glass replication exhibits high temperature stability, serving as a template for the metal casting and hot embossing of amorphous metals. This process enables the rapid and cost-efficient production of sub-micron-structured tooling.

*Sebastian Kluck, Richard Prediger, Leonhard Hambitzer, Manuel Luitz, Niloofar Nekoonam, Franziska Dreher, Markus Lunzer, Matthias Worgull, Marc Schneider, Bastian E. Rapp, Frederik Kotz-Helmer^*^*

**Sub-Micron Replication of Fused Silica Glass and Amorphous Metals for Tool-based Manufacturing**


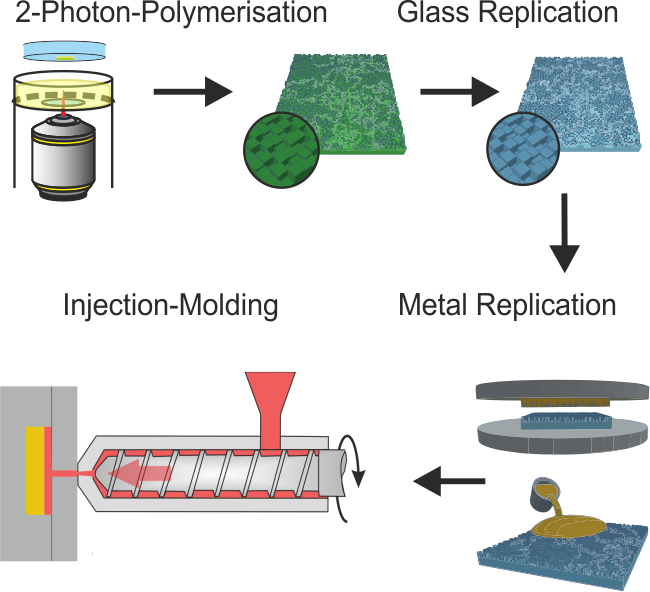

Supplement: Supplementary file 1 — Supporting Information [file ADVS-11-2405320-s001.docx]
